# Genetic covariance analysis of Alzheimer's disease and stroke implicates *PHLPP1* as a shared locus in individuals of African ancestry

**DOI:** 10.1002/alz70855_107411

**Published:** 2025-12-25

**Authors:** Nicholas R. Ray, Ajneesh Kumar, Brian W Kunkle, Farid Rajabli, William S Bush, Liyong Wang, Wanying Xu, Fulai Jin, Elanur Yilmaz, Caghan Kizil, Luciana Bertholim Nasciben, Sofia Moura, Aura M Ramirez, Rufus O Akinyemi, Adesola Ogunniyi, Olusegun Baiyewu, Anthony J Griswold, Motunrayo Coker, Kyle M Scott, Kazeem Akinwande, Larry D Adams, Samuel Diala, Patrice G Whitehead, Jacob L. McCauley, Mayowa Ogunronbi, Kara L Hamilton‐Nelson, Albertino Damasceno, Andrew F Zaman, David Ndetei, Susan H. Blanton, Albert Akpalu, Michael L Cuccaro, Kolawole Wahab, Katalina F. McInerney, Karen Nuytemans, Reginald Obiako, Pedro R Mena, Njideka U Okubadejo, Izri M Martinez, Raj Kalaria, Jonathan L Haines, Giuseppe Tosto, Scott M Williams, Allison M Caban‐Holt, Goldie S Byrd, Jeffery M Vance, Margaret Pericak‐Vance, Christiane Reitz, AfDC

**Affiliations:** ^1^ Columbia University Irving Medical Center, New York, NY, USA; ^2^ University of Miami Miller School of Medicine, Miami, FL, USA; ^3^ Case Western Reserve University School of Medicine, Cleveland, OH, USA; ^4^ College of Medicine, University of Ibadan, Ibadan, Oyo, Nigeria; ^5^ Faculty of Medicine, Eduardo Mondlane University, Maputo, Mozambique; ^6^ University of Ghana Medical School, Accra, Ghana; ^7^ Department of Medicine, University of Ilorin, Ilorin, Nigeria; ^8^ Ahmadu Bello University, Zaria, Kaduna, Nigeria; ^9^ College of Medicine, University of Lagos, Lagos, Nigeria; ^10^ Institute for Ageing and Health, Newcastle University, Newcastle, United Kingdom; ^11^ Wake Forest University School of Medicine, Winston‐Salem, NC, USA

## Abstract

**Background:**

Neuropathological and neuroimaging studies indicate that cerebrovascular disease (CVD) is a major risk factor for Alzheimer's disease (AD), yet the molecular mechanisms underlying the association between the two traits remain unclear. To elucidate the mechanistic relationship between the two phenotypes, the current study examined the genetic correlation between stroke and AD in individuals of African ancestry.

**Method:**

Capitalizing on the results from recent genome‐wide association studies (GWAS) on AD (2,844 cases; 6,521 controls) and stroke (3,961 cases; 20,030 controls) in individuals of African ancestry, genetic correlation analysis was conducted using LAVA, which partitions the genome based on LD structure and estimates local genetic covariance within each resulting partition. Enhanced Hi‐C Capture Analysis (eHiCA) was performed at identified top loci to examine chromatin interactions using 8 AD brain autopsy samples from different ancestries and iPSC‐derived cells. In addition, overlapping top loci were examined in 553 African individuals with WGS data from the READD‐ADSP.

**Result:**

Genetic covariance analysis identified a locus shared between AD and stroke on chromosome 18q21.33 that includes the *PHLPP1* gene (ρ = .77, *p* = 2.41×10^‐6^). Examination of the LD structure and genetic association patterns at this locus identified disease‐associated haplotypes exerting an effect in the same direction in both traits. eHiCA demonstrated that the two haplotypes at *PHLPP1* interact with each other, and both haplotypes interact with the same chromosome regions at and around *PHLPP1*. A similar pattern of chromatin interactions, which indicates a coordinated regulation of the same set of genes, was observed in brains across ancestries, and in different AD‐relevant cell types (neurons, microglia, and oligodendrocytes). Variants at *PHLPP1* were also nominally significant in the independent meta‐analysis of African individuals that included WGS data (*p* = 4.56 × 10^‐5^).

**Conclusion:**

These findings nominate *PHLPP1* as a shared locus for both AD and stroke in individuals of African ancestry. Notably, a genome‐wide significant signal in this locus was identified in an admixture mapping study of African Americans (Rajabli et al., 2023). Identification of shared molecular mechanisms between AD and CVD in different populations is useful for understanding the relationship between these two processes in all people.

